# A multisubstrate reductase from *Plantago major*: structure-function in the short chain reductase superfamily

**DOI:** 10.1038/s41598-018-32967-1

**Published:** 2018-10-04

**Authors:** Rachel Fellows, Christopher M. Russo, Catarina S. Silva, Soon Goo Lee, Joseph M. Jez, John D. Chisholm, Chloe Zubieta, Max H. Nanao

**Affiliations:** 1 European Synchrotron Radiation Facility, Structural Biology Group, 71 Avenue des Martyrs, F-38000 Grenoble, France; 2 Department of Chemistry, Syracuse University, Syracuse, NY 13244 USA; 3 Department of Biology, Washington University in St. Louis, One Brookings Drive, Campus Box 1137, St. Louis, MO 63130 USA; 4 Laboratoire de Physiologie Cellulaire & Végétale, Univ. Grenoble Alpes, CNRS, CEA, INRA, BIG, Grenoble, USA

## Abstract

The short chain dehydrogenase/reductase superfamily (SDR) is a large family of NAD(P)H-dependent enzymes found in all kingdoms of life. SDRs are particularly well-represented in plants, playing diverse roles in both primary and secondary metabolism. In addition, some plant SDRs are also able to catalyse a reductive cyclisation reaction critical for the biosynthesis of the iridoid backbone that contains a fused 5 and 6-membered ring scaffold. Mining the EST database of *Plantago major*, a medicinal plant that makes iridoids, we identified a putative 5β-progesterone reductase gene, *PmMOR* (*P*. *major multisubstrate oxido-reductase*), that is 60% identical to the iridoid synthase gene from *Catharanthus roseus*. The PmMOR protein was recombinantly expressed and its enzymatic activity assayed against three putative substrates, 8-oxogeranial, citral and progesterone. The enzyme demonstrated promiscuous enzymatic activity and was able to not only reduce progesterone and citral, but also to catalyse the reductive cyclisation of 8-oxogeranial. The crystal structures of PmMOR wild type and PmMOR mutants in complex with NADP^+^ or NAD^+^ and either 8-oxogeranial, citral or progesterone help to reveal the substrate specificity determinants and catalytic machinery of the protein. Site-directed mutagenesis studies were performed and provide a foundation for understanding the promiscuous activity of the enzyme.

## Introduction

The SDR superfamily has been identified in organisms from viruses to higher eukaryotes, constituting an ancient lineage retained in all kingdoms of life^1^. Members of the SDR superfamily adopt a Rossmann-fold and use NADH or NADPH as a hydride source for the reduction of diverse substrates, most often carbonyl or alkene moieties that are reduced to the corresponding alcohol or alkane. A subgroup of the SDR family found in plants comprises the 5ß-progesterone oxido-reductases (5ß–POR) and the iridoid synthases and has been termed the PRISE family for (**p**rogesterone-5β-**r**eductase and/or **IS** activity displaying **e**nzymes)^2^, although the substrate specificity of the enzymes in this group is likely much broader than progesterone or iridoid precursors^3^.

**Figure 1:**
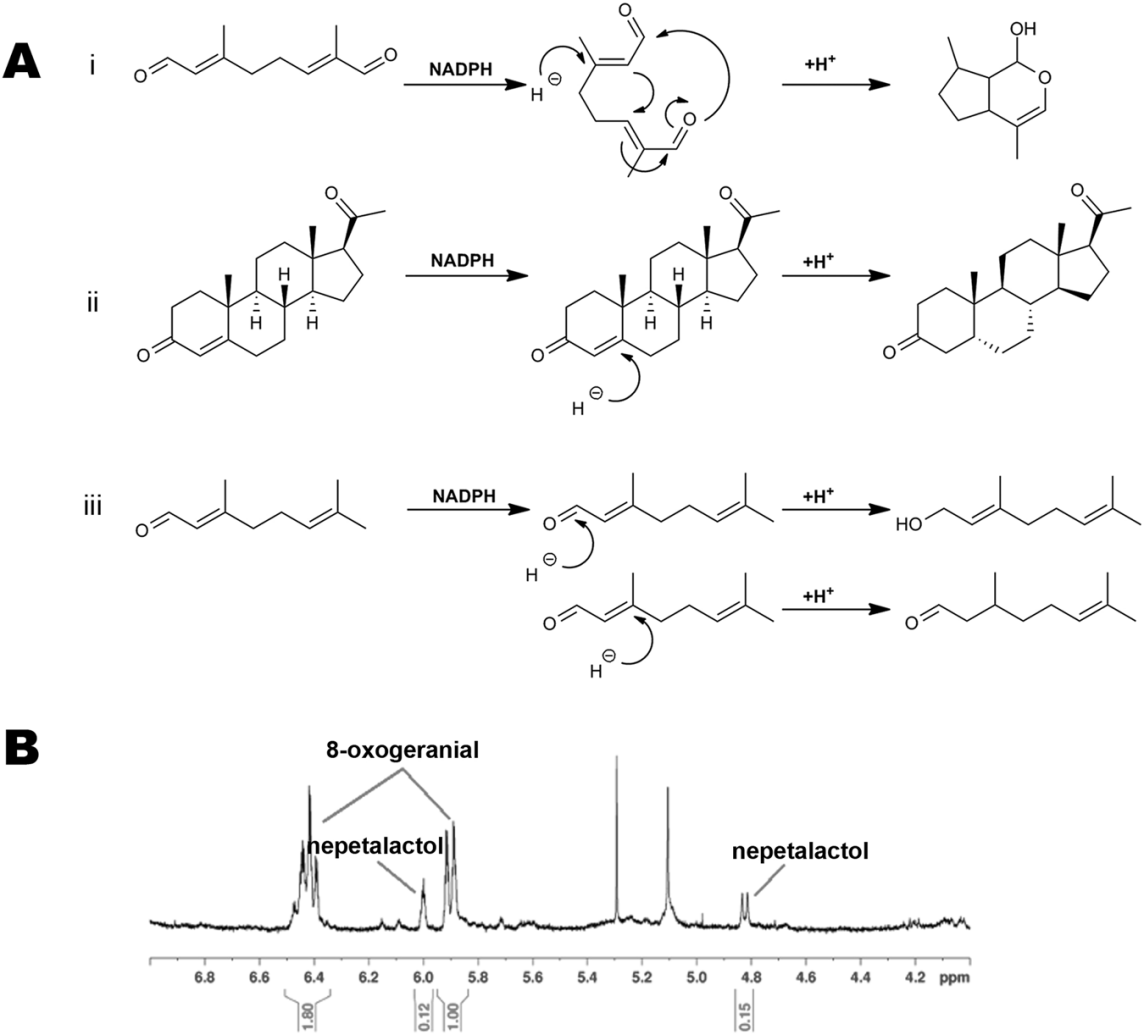
PRISE family reduction reactions. (**A**) Substrates and products for NADPH-dependent reduction reactions for PmMOR, CrIS and Dl5ß –POR. (i) Reductive cyclisation reaction of 8-oxogeranial postulated for CrIS and PmMOR. (ii) Double bond reduction reaction at the 5-position of the A ring of progesterone catalyzed by Dl5ß –POR. (iii) Reduction of the aldehyde (top) and alkene (bottom) of citral by PmMOR yielding alcohol and reduced products. (**B**) ^1^H NMR spectrum of an overnight reaction of PmMOR with NADPH and 8-oxogeranial. The chemical shifts and coupling patterns correspond to the starting material, 8-oxogeranial or nepetalactol and are indicated.

Progesterone reductases and iridoid synthases share a high degree of sequence and structural homology, but perform distinct reactions and act in different biosynthetic pathways^4^. Progesterone is derived from the cardenolide pathway found in species such as *Digitalis lanata* and *Erysimum crepidifolium*^5^. Based on sequence conservation, putative 5β-PORs have been identified in over 100 plant species, including many angiosperms that do not make cardenolides^6^. Thus, while demonstrating a high degree of primary, secondary and even tertiary structure conservation, the 5β-PORs likely have more diverse *in vivo* functions than the reduction of progesterone. However, in the majority of cases, their true substrates have not been unambiguously identified. Unlike the 5ß–PORs that perform classical reduction reactions, the IS enzymes catalyze a reductive cyclisation to form the fused 5 and 6-membered ring of the iridoid backbone^4^ (Fig. 1). IS enzymes have been identified and well characterized in periwinkle and olive and act as the committing branch point enzyme in iridoid biosynthesis^4,7^. Indeed, iridoid-derived compounds are much more common plant natural products than cardenolides, further suggesting that many 5β-PORs are misannotated and act on different substrates, including 8-oxogeranial, the iridoid scaffold precursor. Due to the medicinal importance of iridoid and iridoid-derived compounds^8–15^, the elucidation of the pathway responsible for iridoid biosynthesis is under extensive and active study^4,7,13,16–19^. Current research suggests that many of the iridoid pathway enzymes are conserved in the angiosperm lineage and that the synthesis of iridoid compounds is very widespread in plants^3^.

*Plantago major* is a medicinal plant with a world-wide distribution that produces an extensive array of iridoid derivatives which are known to be bioactive and is thus an attractive target for investigating iridoid biosynthesis^20^. *P*. *major* does not have the cardenolide pathway, however it does have an annotated 5β-POR. In order to investigate the function of this protein, which we termed PmMOR (*Plantago major* multisubstrate oxido-reductase), we expressed, purified and determined its activity with different substrates including 8-oxogeranial, citral and 5β-progesterone. In order to probe the substrate specificity determinants and enzymatic mechanism, we further solved the crystal structures of PmMOR as a ternary complex with NADP^+^ and 8-oxogeranial, PmMOR in complex with NAD^+^ and progesterone, PmMOR^V150M^ in complex with NADP^+^ and citral, and PmMOR^V150M^ in complex with NADP^+^ and progesterone. Comparing the results for PmMOR with the PRISE family members, *C*. *roseus* iridoid synthase (CrIS) and *Digitalis lanata* 5ß–POR (Dl5ß–POR), offers new insight into the substrate specificity determinants and catalytic activity of the PRISE enzyme family and suggests that these enzymes are able to act on diverse substrates and perform both reduction and reductive cyclisations.

## Results

### Identification of PRISE family genes from *P*. *major* and protein expression

Based on sequence conservation with *CrIS* (GB JX974564) and *Dl5ß–POR* (GB AIF73578.1) we searched GenBank for putative PRISE family members from *P*. *major* using PSI-BLAST^21^. Although a complete genome of *P*. *major* is not yet available, an EST database is accessible and a gene annotated as a 5ß–POR was identified (ADG56541.1) with an E value of 3e^−164^ based on sequence identity to CrIS, with no other genes with significant homology in the search results^22^. This is consistent with the high degree of sequence similarity between CrIS and 5ß–PORs from other plants^4^. Given the known production of iridoids, the lack of a cardenolide pathway in *P*. *major*, and that some 5ß–PORs have been shown to also have IS activity^23^, we therefore reasoned that the gene annotated as a 5ß–POR from *P*. *major* could in fact possess IS activity. Based on the translated amino acid sequence of the *P*. *major* ADG56541.1 gene, here termed *PmMOR* for *Plantago* *major* multisubstrate oxido-reductase, we designed an expression construct with an encoded N-terminal poly-histidine tag, TEV cleavage site and optimized for *E*. *coli* codon usage (see Supplemental Fig. 1 for sequence). Recombinant overexpression of the synthetic gene yielded over 5 mg of protein per liter of cells. Purification to homogeneity of the protein was performed using metal affinity chromatography with nickel-affinity resin and size-exclusion chromatography. The protein exhibited a strong absorbance at 220 nm, indicative of binding of a dinucleotide cofactor. Based on the elution volume, the protein was dimeric in solution.

### Wild-type enzymatic activity assays and NMR

The activity of the protein was assayed using 8-oxogeranial (the substrate for iridoid biosynthesis), citral (a commercially available mixture of geranial and neral which are substrate mimetics) and progesterone (Fig. 1). The reaction was monitored spectrophotometrically via the consumption of NADPH. The enzyme demonstrated the ability to reduce the two substrates, citral and 8-oxogeranial, with similar turnover number; however, the *K*_m_ was higher for 8-oxogeranial (368 μM) than it was for citral (82 μM), even though the substrates have very similar chemical structures (Table 1).

**Table 1:** PmMOR enzymatic activity.

	*V*_max_ (nmol/min)	*K*_m_ (µM)	*k*_cat_ /*K*_m_ (M^−1^sec^−1^)
PmMOR Citral	1.52 +/− 0.028	82 +/− 4.5	1.7 × 10^4^
PmMOR progesterone	0.14 +/− 0.02	28 +/− 3.0	2.1 × 10^3^
PmMOR 8-oxogeranial	5.3 +/− 0.32	368 +/− 37	2.6 × 10^3^

To confirm that PmMOR catalyzed the reductive cyclisation of 8-oxogeranial and not only reduction of the aldehyde, a large-scale reaction using a NADPH regeneration system was used to produce enough material for ^1^H NMR analysis according to published protocols^4^. This sample was then analyzed to confirm the production of a cyclised product. ^1^H NMR spectra of the starting material, 8-oxogeranial and nepetalactol, the cyclised reaction product, were recorded and compared with the crude product extract. Peaks corresponding to both unreacted 8-oxogeranial and nepetalactol (major product) were observed in the NMR spectrum, as well as additional small peaks corresponding to a potential secondary unknown product (Fig. 1B). While nepetalactol could be unambiguously identified, the secondary product could not be purified in sufficient quantities for characterization, but likely represents side reactions yielding alcohol and/or alkene reduction products.

### Three-dimensional structure of PmMOR

**Figure 2:**
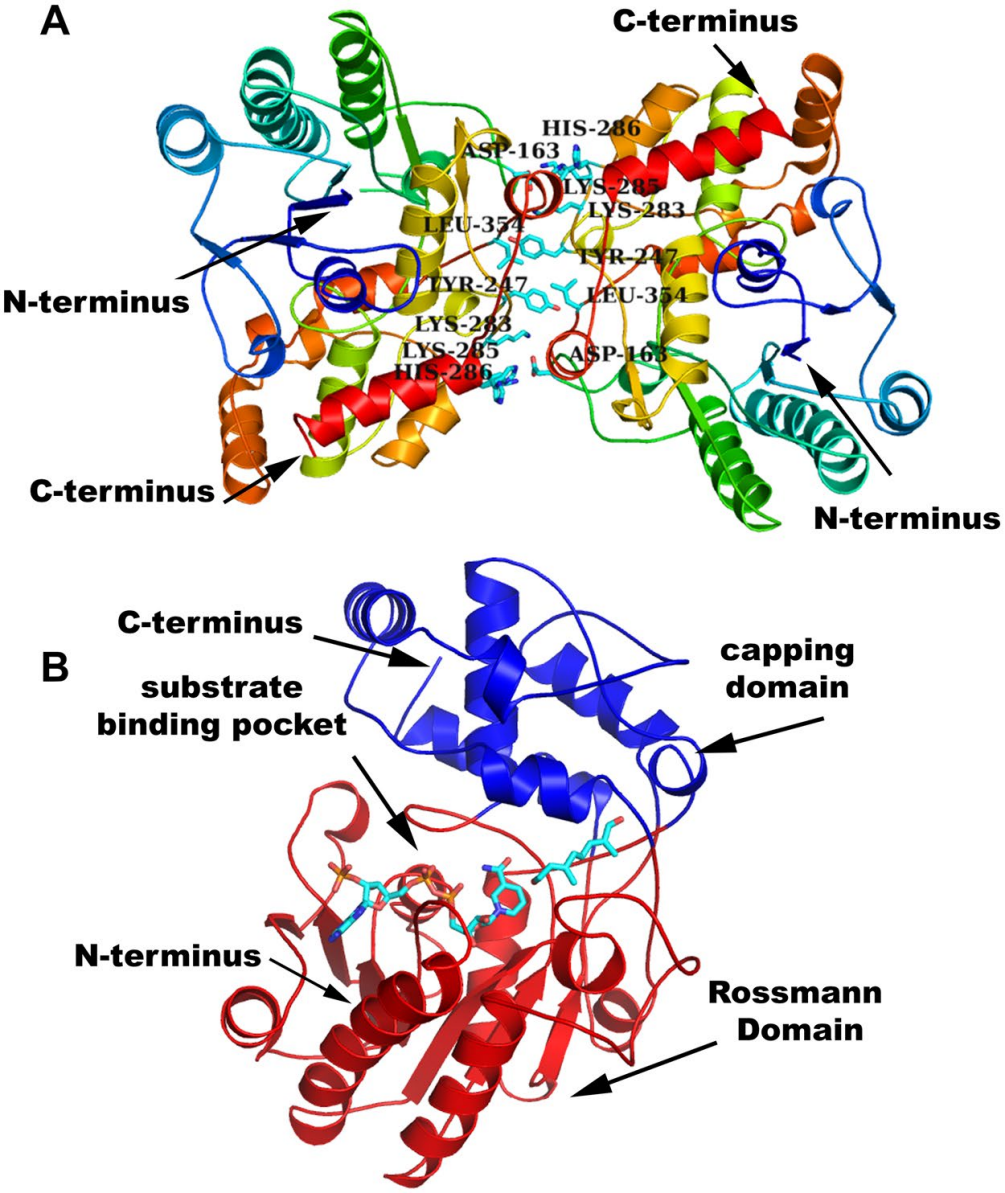
Overall fold of PmMOR. (**A**) PmMOR dimer shown as cartoon and colored as rainbow from N (blue) to C (red)-terminus, termini are labeled. Residues involved in dimerisation are labeled, shown as sticks and colored by atom with carbons in cyan. (**B**) PmMOR cartoon bound with NADP^+^ and 8-oxogeranial shown as sticks with carbons in cyan and colored by atom. The capping domain is shown in blue and the Rossmann domain in red. Domains and substrate binding pocket are indicated with arrows and termini are labeled.

To further characterize PmMOR, we determined the crystal structure of the wild-type enzyme in complex with 8-oxogeranial and NADP^+^, progesterone and NAD^+^ and the crystal structure of the point mutant V150M (referred to as PmMOR^V150M^) in complex with NADP^+^ and citral or progesterone (Table 2). There were no major structural rearrangements with the different substrates and substrate mimetics. The overall fold is highly conserved with the previously described 5ß–POR from *D*. *lanata*^24^ and the iridoid synthase from *C*. *roseus*^25–27^. All three enzymes are dimeric in solution as determined by size-exclusion gel purification and the respective crystal structures. The conserved dimerisation interface of PmMOR buries approximately 7360 Å^2^ of surface area, corresponding to approximately 25% of the total surface area of the dimer as determined using the PDBePISA server^28^ (Fig. 2A). Based on sequence alignments, the residues involved in dimerisation are highly conserved between the proteins with salt bridges (ASP-163 and LYS-283, ASP-163 and LYS-285, ASP-163 and HIS-286), hydrogen bonds (TYR-247 and the amide backbone of LEU-354), and extensive hydrophobic interactions contributing to the dimerisation interface (Fig. 2A). While the *D*. *lanata* 5ß–POR structure was lacking the N-terminal 13 residues and the CrIS structures were truncated in the N-terminal region (25 residues for 5COA, 5COB, 5DBF and 22 residues for 5DCW, 5DCU, 5DCY, 5DF1), the PmMOR protein in all our experiments is full-length (Fig. 2B). Previous reports suggested that the *D*. *lanata* 5ß–POR N-terminal region might have a role in oligomerisation; however, in the PmMOR structure these residues do not contact the dimerisation interface and are thus unlikely to be involved in higher order oligomer formation^24,29^.

**Table 2:** Data collection and refinement statistics.

	8-oxogeranial/NADP^+^ WT	Citral/NADP^+^ PmMOR^V150M^	Progesterone/NADP^+^ PmMOR^V150M^	Progesterone/NAD^+^ WT
Wavelength, Å	0.8726	0.8726	0.8726	0.8726
Resolution range, Å	31.6–1.86 (1.93–1.86)	28.1–1.46 (1.516–1.463)	39.2–2.56 (2.66–2.56)	39.04–2.7 (2.80–2.7
Space group	*P*4_3_2_1_2	*P*4_3_2_1_2	*P*4_3_2_1_2	*P*4_3_2_1_2
Unit cell, a,b,c in Å and α,β,γ in°	79.5, 79.5, 138.2 90, 90, 90	79.5, 79.5, 137.6 90, 90, 90	78.3, 78.3, 135.9 90, 90, 90	78.8,78.8, 134.8, 90, 90, 90
# Total reflections^*^	489693 (41373)	524364 (42849)	117760 (12168)	89723 (9221)
# Unique reflections^*^	37909 (2981)	76269 (7239)	14183 (1085)	11722(1142)
Multiplicity^*^	12.9 (11.2)	6.9 (5.9)	8.3 (8.7)	7.7 (8.1)
Completeness, %^*^	89 (99)	99 (96)	98 (100)	95.5 (97.5)
Mean I/sigma(I)^*^	27.6 (1.8)	17.1 (1.6)	18.3 (2.1)	16.3 (1.62)
Wilson B-factor, Å^2^	37.1	18.3	57.3	79.9
R_merge_^*^	0.055 (1.41)	0.056 (1.03)	0.090 (0.951)	0.080 (1.20)
R_meas_^*^	0.057 (1.48)	0.060 (1.13)	0.096 (1.01)	0.087 (1.29)
CC^1/2*^ (%)	100 (71.2)	99.9 (57)	99.9 (84)	99.9 (42.6)
# Reflections used in refinement^*^	33916 (2980)	76265 (7239)	13860 (1085)	11704 (1142)
# Reflections used for R-free^*^	1698 (156)	3834 (323)	700 (52)	552 (56)
R_work_^*^	0.167 (0.383)	0.151 (0.265)	0.178 (0.325)	0.204 (0.312)
R_free_^*^	0.211 (0.436)	0.171 (0.275)	0.258 (0.364)	0.261 (0.391)
Number of non-hydrogen atoms	3203	3552	3043	3010
macromolecules	2943	2954	2912	2917
ligands	68	73	71	67
Protein residues	364	364	364	364
RMS bonds, Å	0.020	0.013	0.008	0.020
RMS angles, °	1.82	1.55	0.97	1.40
Ramachandran favored; allowed; outlier (%)	92.5; 7.5; 0	94.3; 5.7; 0	89.3; 10.7; 0	91.5; 8.5; 0
Rotamer outliers (%)	3.7	0.61	3.8	4.4
Clashscore	1.52	2.53	7.21	6.99
Average B-factor (Å^2^)	49.3	26.8	60.9	85.2
protein	49.1	24.5	61.2	85.3
ligands	47.6	19.8	58.0	86.5
solvent	52.6	40.5	50.0	68.3

^*^Statistics for the highest-resolution shell are shown in parentheses.

**Figure 3:**
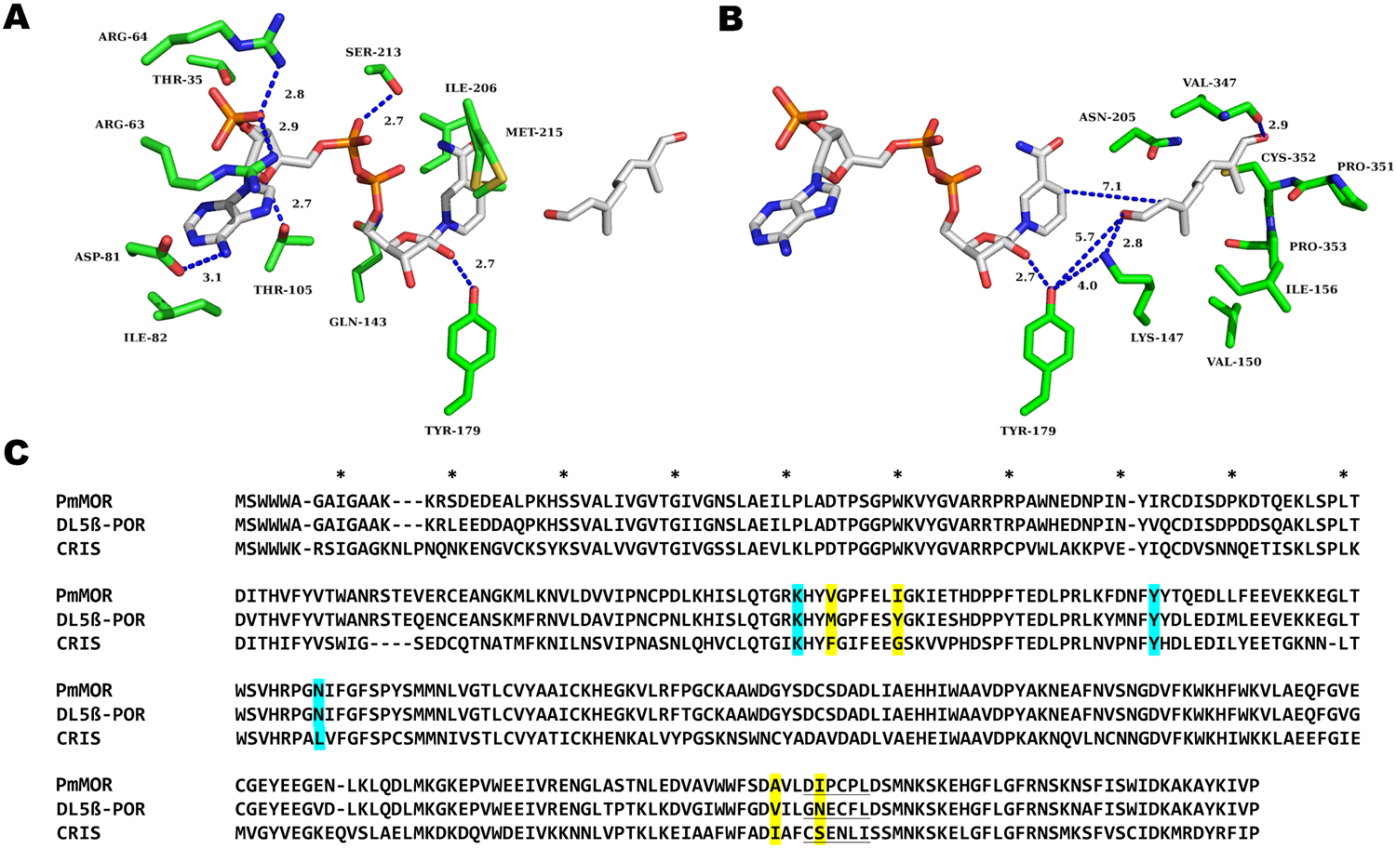
Active site close-up of PmMOR bound with NADP^+^ and 8-oxogeranial and sequence alignment of PmMOR, CrIS and Dl5ß-POR. (**A**) NADP^+^ binding residues depicted as sticks. Residues are labeled and hydrogen bonding interactions are depicted as dotted blue lines with the distances between atoms given. The cofactor binding site is formed primarily by the Rossmann fold domain. Residues ILE-206, MET-215 and SER-213 from the helical cap domain help to sequester the cofactor. (**B**) 8-oxogeranial binding site labeled as per (**A**). The distance between the reactive hydride and the substrate is 7.1 Å, non-optimum for catalysis. (**C**) Sequence alignment of *P*. *major* multifunctional oxido-reductase (PmMOR), *D*. *lanata* 5-ß progesterone oxido-reductase (Dl5ß-POR) and *C*. *roseus* iridoid synthase (CrIS). Approximately every tenth position is marked with an asterisk. Putative catalytic residues are highlighted in cyan and active site mutants are highlighted in yellow. The loop region that differs in orientation between PmMOR and CrIS corresponding to residues 349–354 is underlined.

Each monomer of the protein folds into two domains, a Rossmann domain that binds NADP(H) and forms much of the active site cavity and a largely α-helical capping domain. The Rossmann domain has a characteristic β-α-β motif with the seven β-strands forming a parallel β-sheet. The capping domain, formed via an insertion between β-strands 6–7 of the Rossmann fold and the C-terminal residues, encloses the cofactor binding site and contains the majority of the 8-oxogeranial substrate binding residues (Figs 2B and 3A). Comparisons of the overall primary, secondary and tertiary structures between PmMOR, CrIS, and Dl5ß–POR reveal a high degree of conservation. PmMOR and CrIS share 60% sequence identity and a RMSD of 1.0 Å over 366 residues. PmMOR and Dl5ß –POR share 87% sequence identity over 363 residues and a RMSD of 0.71 Å^30^ (Fig. 3C). While the overall fold is highly conserved, the residues in the region that interacts with the substrates are less well conserved, accounting for the different substrate specificity and activity of the enzymes.

### Cofactor binding site

The majority of residues that interact with NADP(H) are contributed from the Rossmann domain and are completely conserved between PmMOR, CrIS and 5ß–POR from *D*. *lanata* (Fig. 3C). An extensive hydrogen bonding network positions the NADP(H) molecule in the active site. ASP-81hydrogen bonds to the exo-cyclic amine of the adenine ring and the N1 nitrogen of the adenine moiety interacts with the backbone amide of ILE-82. ARG-64 and ARG-63 bind to the adenosine ribose phosphate moiety, the THR-35 backbone amide and carbonyl interacts with the ribose hydroxyl and THR-105 hydrogen bonds with the ribose cyclic oxygen. The diphosphate is positioned via interactions with the side chain of SER-213 and the backbone amide of ILE-37. The nicotinamide ribose oxygen hydrogen bonds to GLN-143 and the 3′-hydroxyl to the sidechain of TYR-179. The nicotinamide moiety is positioned via interactions with the backbone carbonyl and amide of MET-215 and ILE-206, respectively (Fig. 3A).

### Substrate binding site

**Figure 4:**
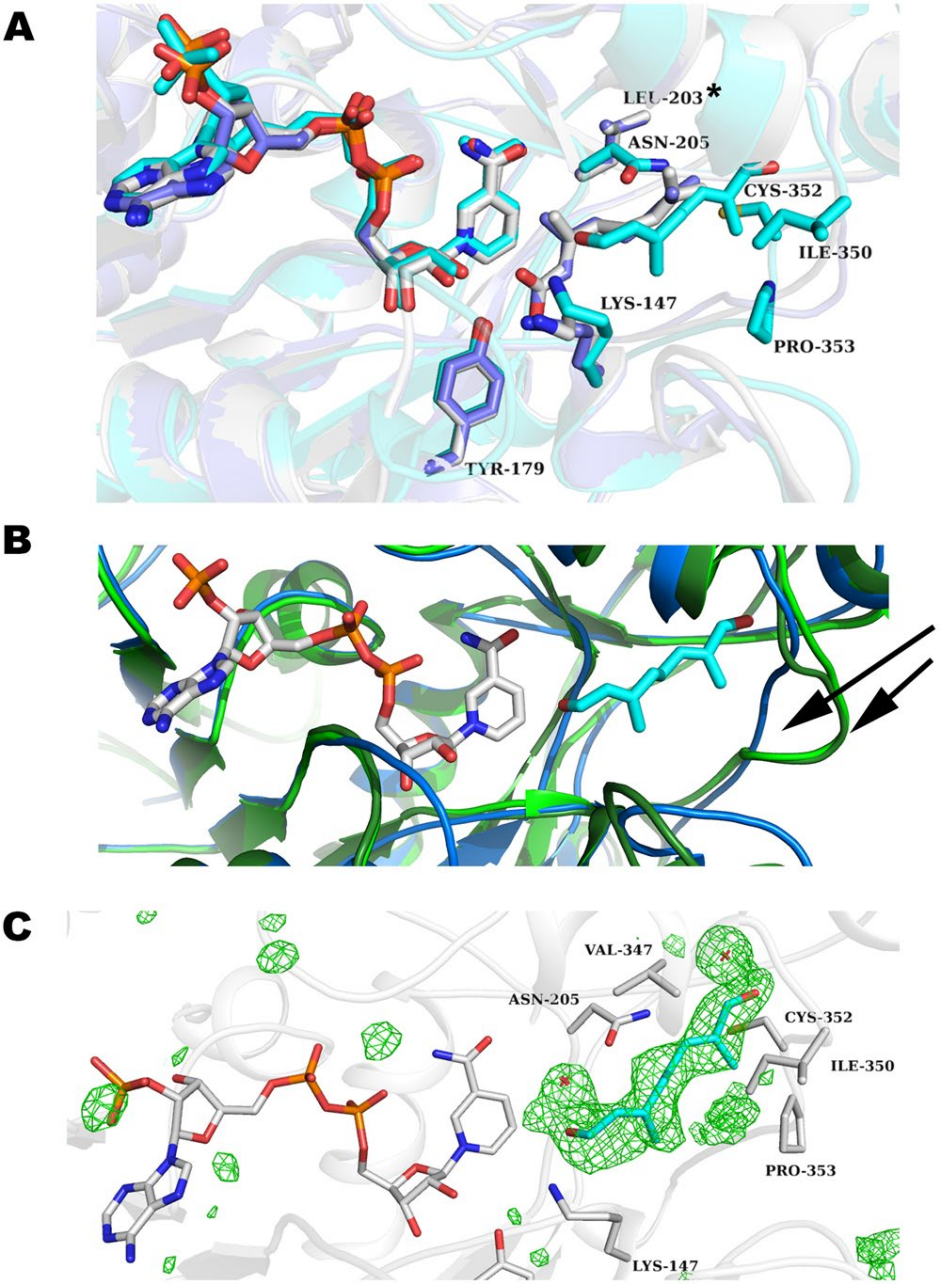
Ligand binding pocket comparison. (**A**) Close-up view of the 8-oxogeranial binding site of PmMOR (carbons colored cyan) overlaid with 5DF1 (CrIS with NADP^+^ and geranic acid with carbons colored gray) and 5DBI (CrIS with NADP^+^ and 8-oxogeranial with carbons colored purple). Residues are labeled as per PmMOR numbering except LEU-203, which corresponds to CrIS. 8-oxogeranial is shifted away from the NADP^+^ in PmMOR versus CrIS. (**B**) Active site view highlighting the shifted loop between PmMOR (dark blue) and CrIS (dark and light green). The 349-DIPCPL-354 loop is indicated with black arrows. (**C**) Omit map contoured at +3.5 standard deviations (σ) above the mean shown in green mesh for the PmMOR/NADP^+^/8-oxogeranial structure. Selected side chain residues are drawn as sticks and labeled.

The 8-oxogeranial binding site is at the interface of the two domains with most substrate sequestering residues contributed from the capping domain and not the Rossmann domain with distances given for catalytic and hydrogen bonding residues (Fig. 3B). As seen in the ligand complexes of CrIS^25–27^ and PmMOR (this work), the substrate-binding pockets of CrIS and PmMOR are shaped differently (Fig. 4A,B), and indeed PmMOR is able to accommodate diverse substrates, unlike CrIS, which is consistent with the ability of PmMOR to reduce progesterone, citral and 8-oxogeranial. The PmMOR 8-oxogeranial complex structure reveals a somewhat different binding mode compared to the CrIS structures^25,26^ in which the ligand is shifted away from the cofactor (Fig. 4A). This shift is likely due to the extended PmMOR active site and several residues that differ between CrIS and PmMOR. The most notable examples of the latter include PmMOR ASN-205 (CrIS LEU-203), which points directly into the active site and is incompatible with the CrIS 8-oxogeranial binding mode, as well as PmMOR VAL-347 (CrIS ALA-346) and PmMOR ILE-350 (CrIS SER-349) (Fig. 4A,C). Finally, the loop containing residues ASP-349 to LEU-354 (DIPCPL in PmMOR vs CSENLI in CrIS) is in a different conformation in the PmMOR compared to the CrIS structures and forms part of the binding surface for 8-oxogeranial in PmMOR and is in a different conformation in the CrIS structures (Fig. 4B,C).

**Figure 5:**
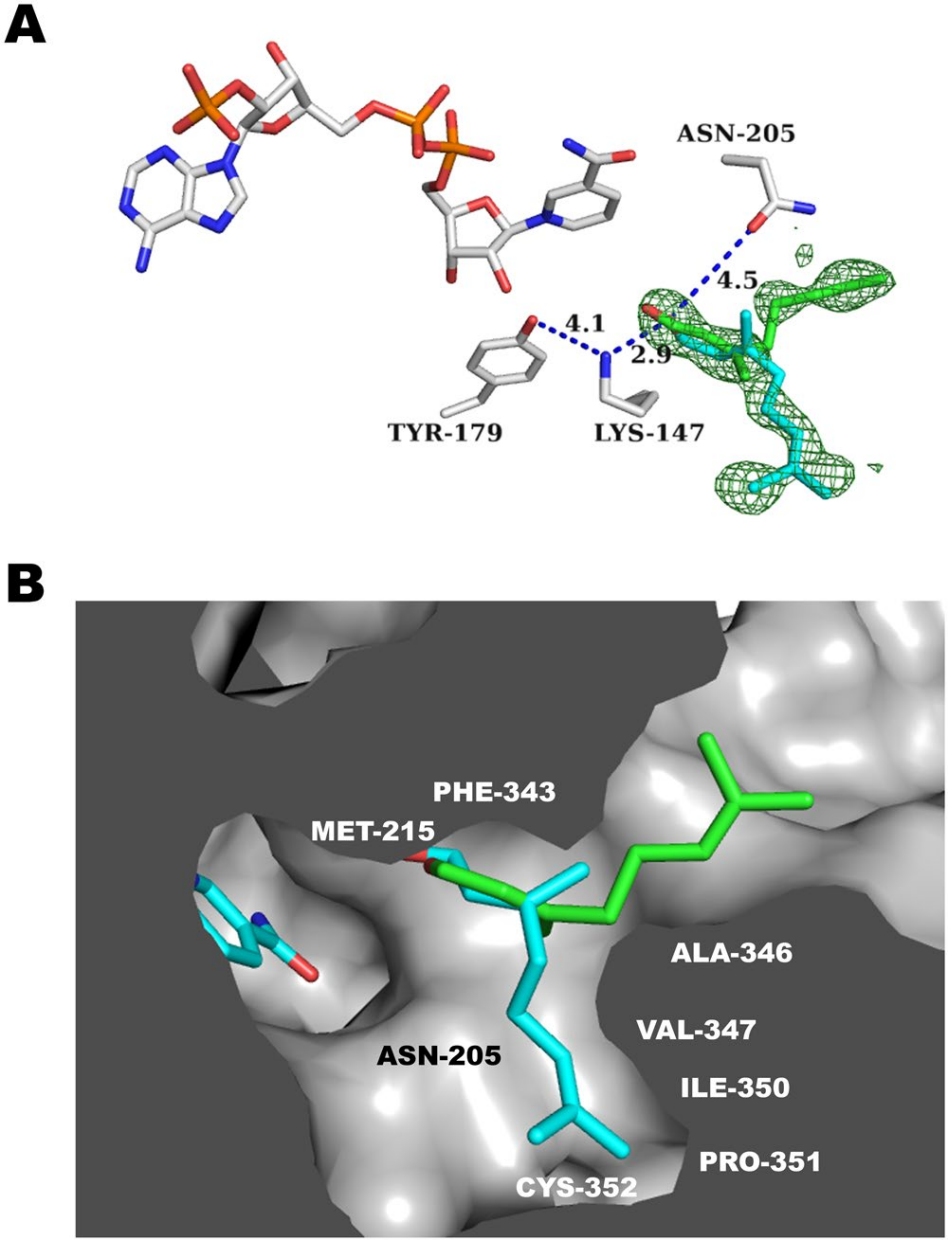
PmMOR^V150M^ citral binding. (**A**) Close-up view of the active site showing putative catalytic residues for citral with distances shown as blue dashed lines and labeled. The two citral conformations are show with carbons in cyan and green. Omit map contoured at +3.5 standard deviations (σ) above the mean shown in green mesh. (**B**) A cutaway of the PmMOR active site is shown with the two citral binding modes colored as per A. Selected residues are labeled.

Initial attempts to determine the complex structure with citral produced diffuse and poor electron density in the active site. None of these datasets yielded interpretable electron density for the ligand. We were, however, able to obtain higher quality ligand electron density using the PmMOR^V150M^ mutant instead of wild type. PmMOR^V150M^ replaces a small hydrophobic residue in the active site substrate-binding pocket with a larger residue, which is present in Dl5ß–POR (Fig. 3C). We reasoned that this change in the active site might lead to better quality electron density for the substrate molecules by restricting the size of the ligand binding site. In the PmMOR^V150M^/NADP^+^/citral structure, the hydrophobic citral substrate is found in two distinct conformations, one of which is similar to the binding mode observed in the PmMOR 8-oxogeranial structure and a second conformation in which the molecule is flipped downwards into the active site (Fig. 5). This secondary conformation could be due to the isomeric mixture of citral (geranial and neral), but the electron density was not of sufficient quality to unambiguously distinguish between the two possibilities (Fig. 5A). As citral lacks the terminal aldehyde, it may not be as well anchored as 8-oxogeranial and the molecule is therefore able to sample a greater diversity of binding modes within the large substrate pocket. The reactive hydride of the NADPH molecule would be positioned approximately 5–6 Å from the aldehyde of citral, leading to reduction of the aldehyde to the corresponding alcohol. The double bond at the C2/C3 position in citral is approximately 8 Å from the reactive hydride, a non-optimum distance for hydride transfer. However, based on enzymatic assays and TLC analysis, two products were observed in the reaction. Thus, it is likely that the enzyme is able to form both reduction products. The 1.86 Å crystal structure of the ternary complex with NADP^+^ and 8-oxogeranial and the 1.46 Å ternary structure with citral shows the 8-oxogeranial molecule in the same relative position as citral with the same residues contributing to substrate sequestration. Thus, it is likely to be a physiologically relevant binding mode.

### Progesterone binding

**Figure 6:**
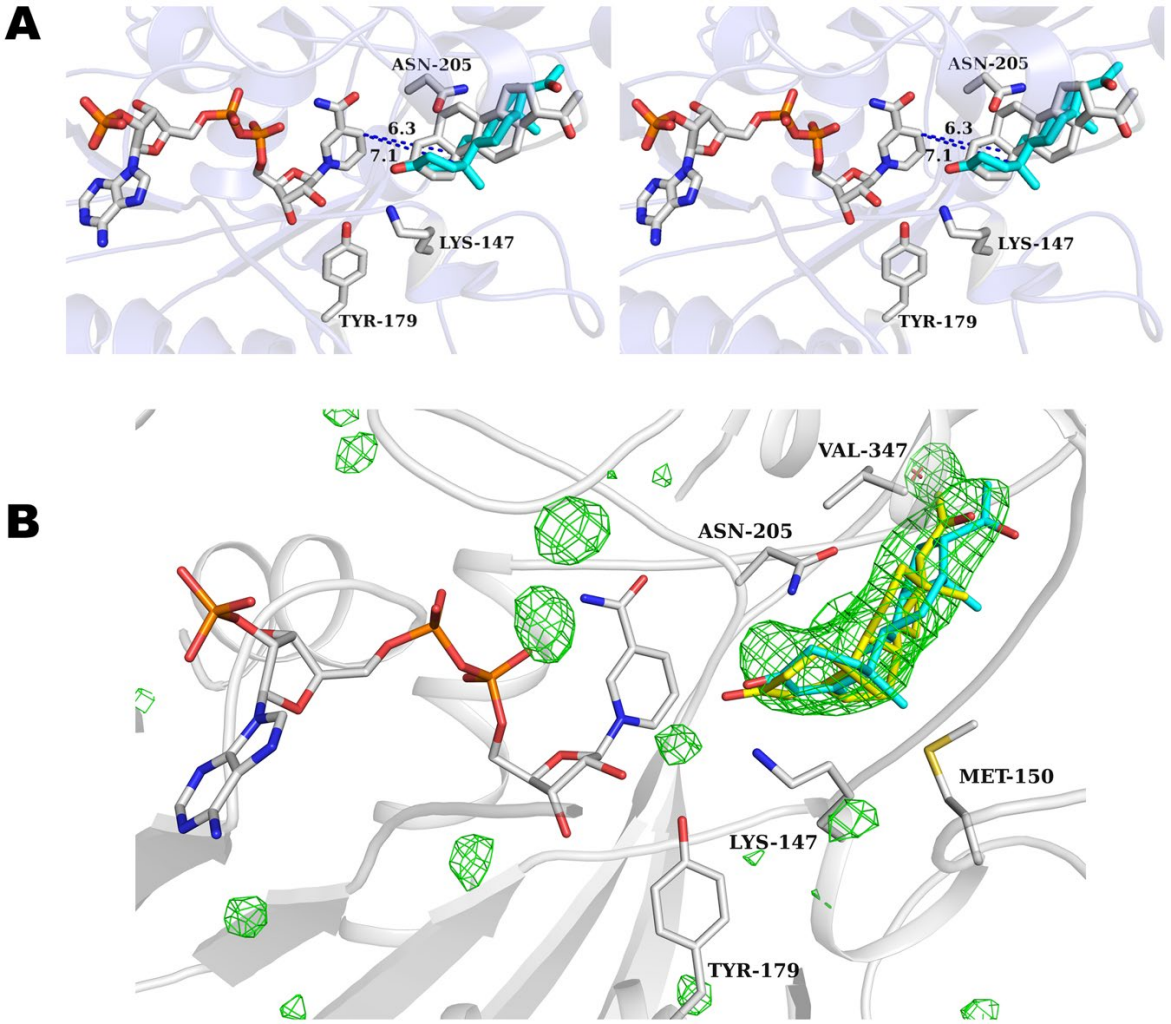
PmMOR^V150M^ progesterone binding. (**A**) Stereo-view of the active site of PmMOR^V150M^ in complex with NADP^+^ and progesterone with protein and side chains shown in gray. Selected residues near the ligand are shown in sticks. NADP^+^ is colored by atom with carbons in gray. Progesterone is colored by atom with carbons in cyan. The binding mode of progesterone postulated by Thorn, *et al*.^24^ is shown and colored by atom with carbons in gray. Note the steric clash with ASN-205 for this binding mode. Distances between the reactive hydride and carbon-carbon double bond to be reduced are 6.3 Å and 7.1 Å for the PmMOR and Thorn, *et al*. conformation, respectively. (**B**) PmMOR^V150M^ progesterone omit map contoured at +3.5 standard deviations (σ) above the mean shown in green mesh. Selected side chain residues are drawn as sticks and labeled. The PmMOR^WT^ progesterone ligand is shown in yellow sticks and the PmMOR^V150M^ progesterone is shown in cyan sticks.

PmMOR shares higher sequence identity to progesterone reductases and may represent a more promiscuous enzyme with greater substrate versatility than either the CrIS or Dl5ß–POR enzymes. To investigate this, we co-crystallized PmMOR^V150M^ with progesterone and NADP^+^. Several datasets were collected which showed significant difference density in the Fo-Fc electron density maps. The V150M point mutation yielded the best results (similar to what we observed for citral binding). Omit maps showed density in the active site, defining the location of the bound steroid (Fig. 6). Progesterone was modeled into this density, however, despite the relatively high resolution of the data (2.56 Å). The resulting density was of poor quality, so the exact position of the ligand should be treated with caution. A second dataset of wild-type protein in complex with progesterone also yielded an extended difference map peak in the same region, into which progesterone could be modeled and refined, however the crystals diffracted to lower resolution and the map was of poorer quality. It is likely that the size of the pocket allows for significant movement of the ligand, and the observed density is an average of multiple conformations. Nevertheless, the binding region presented here of progesterone is largely consistent with the model suggested by Thorn *et al*.^24^. and Hu *et al*.^25^, and places the C3 ketone in the same position as the aldehyde in our 8-oxogeranial and citral bound structures. By contrast, our data and model favor a conformation where the steroid is flipped 180° when compared to the previous models (Fig. 6A)^24,25^. This conformation fits the difference electron density much better as demonstrated by the higher correlation coefficients (CC with the difference map 1.75x higher for the flipped conformation) than the best fit from Thorn *et al*.^24^ and avoids a steric clash with ASN-205 which occludes part of the binding pocket in PmMOR. The conformation is still consistent with the proposed chemical mechanism, but has implications for ligand binding. Examining the environment of the bound progesterone reveals several positions that differ between enzymes with activity for progesterone (PmMOR and Dl5ß –POR) and CrIS, which does not use progesterone as a substrate. PmMOR ARG-146 (ARG in Dl5ß –POR), is an ILE in CrIS, PmMOR ASN-205 (ASN in Dl5ß –POR) is a LEU in CrIS and finally, the loop region containing residues 348–352 have divergent sequences but are structurally conserved between PmMOR and Dl5ß –POR and significantly differ in CrIS (Figs 3C and 6A).

### Site-directed mutagenesis and enzymatic activity of mutants

To probe the residues in the active site important for catalysis and substrate specificity in these highly related enzymes, a series of point mutations of PmMOR was made. As 8-oxogeranial is not commercially available and very limited quantities were available to us, the substrate mimetic citral was used for all mutant assays, as it shows good specificity based on *K*_m_ values for the wild type PmMOR and similar catalytic efficiency to 8-oxogeranial. Specific activity of the mutants was assayed against citral as well as progesterone and compared to Dl5ß –POR.

Based on the structure of PmMOR and comparisons with 5ß–POR (PDB 2V6G), residues putatively important for catalysis and substrate specificity were targeted for site-directed mutagenesis based on their proximity to the bound substrate. Specific activities for the wild type and mutant PmMOR and Dl5ß–POR for different substrates are given in Table 3. Interestingly, the specific activity of PmMOR versus Dl5ß–POR was higher towards both citral and progesterone. While progesterone was poorly utilized as a substrate by PmMOR based on very low specific activity, the enzyme was able to reduce progesterone to 5ß-dihydroprogesterone, even though this compound is not found in *P*. *major* and is thus not a physiological substrate. In addition, PmMOR was able to reduce both 5α-dihydroprogesterone and 5β-dihydroprogesterone whereas no activity towards these compounds was seen for Dl5ß –POR, further highlighting the high degree of substrate promiscuity of PmMOR.

**Table 3:** Specific activities of PmMOR, PmMOR mutants and Dl5ß–POR with progesterone, 5α-dihydroprogesterone, 5β-dihydroprogesterone and citral.

Protein	Specific activity (pmol/min/μg)	Substrate
PmMOR	3.07 ± 0.38	progesterone
PmMOR	2.81 ± 0.12	5α-dihydroprogesterone
PmMOR	2.56 ± 0.23	5β-dihydroprogesterone
Dl5ß–POR	1.79 ± 0.24	progesterone
Dl5ß–POR	no activity	5α-dihydroprogesterone
Dl5ß–POR	no activity	5β-dihydroprogesterone
Dl5ß–POR	27.5 ± 2.86	citral
PmMOR A346V/I350N	2.30 ± 0.18	progesterone
PmMOR V150M/I156Y	7.80 ± 0.97	progesterone
PmMOR V150M	7.67 ± 0.47	progesterone
PmMOR	760 ± 14	citral
PmMOR V150M	883 ± 5.7	citral
PmMOR N205D	5400 ± 5.94	citral
PmMOR V150M/I156Y	1694 ± 52.5	citral

Next we investigated the role of putative catalytic residues in PmMOR. The SDR superfamily generally contains a catalytic tetrad of ASN-SER-TYR-LYS or triad consisting of SER-TYR-LYS; however, the 5ß–PORs lack this canonical tetrad or triad and are thought to use only an active site TYR and LYS residue for catalysis^1,24,31–33^. Residues TYR-179 and LYS-147 are highly conserved in 5ß–PORs, are present in PmMOR and lie between the substrate molecule and NADP^+^ cofactor in the PmMOR structures with distances shown (Fig. 3B,C). ASN-205 is not conserved between 5ß–PORs and ISs. However, ASN-205 is located adjacent to the C6-C7 alkene of citral and 8-oxogeranial in the PmMOR structure, which could potentially be important during the cyclisation reaction. These residues, TYR-179, LYS-147 and ASN-205, based on sequence conservation and/or proximity to the substrate were hypothesized to act as putative catalytic residues during the hydride transfer and reductive cyclisation reactions by providing a proton and/or positioning the enolate anion intermediate for cyclisation and were targeted for mutagenesis studies. While the Y179F mutant did not express and could not be tested for catalytic activity, the N205A, N205D, K147A and K147M mutants all expressed and were purified to homogeneity via nickel-affinity column and size-exclusion chromatography. The N205A mutant exhibited marked substrate inhibition even at low substrate concentrations based on the velocity versus substrate concentration curves and was not further characterized. The N205D mutation increased specific activity, suggesting that while ASN-205 is not critical for the reaction, changes at this position can help tune the rate of reaction due to proximity to the substrate molecule and possible positioning of the enolate intermediate. K147A and K147M mutations completely abrogated enzymatic activity, strongly suggesting that LYS-147 acts as a proton donor during the reduction of the substrate by NADPH either directly to the substrate or via proton transfer to TYR-179 as postulated for Dl5ß–POR^24^. This is in contrast to what has been observed by some groups for CrIS in which little to no differences in activity were seen with lysine mutations versus wild type^26,27^. Reductions to approximately 10–20% of wild type activity were reported in CrIS for lysine to alanine mutations using very similar reaction conditions and protein constructs^25^. In the reported CrIS structures, LYS-148 (CrIS numbering) is between 4.7–5 Å away from the hydroxyl group of the catalytic tyrosine, suggesting a limited role in proton transfer during catalysis. However, the discrepancy in reported enzymatic activities leaves questionable the role of LYS-148 (equivalent to LYS-147 PmMOR) in the CrIS reaction mechanism. In PmMOR, LYS-147 is in a different orientation and only 4 Å away from the hydroxyl group of TYR-179, perhaps accounting for the abrogation of catalytic activity observed due to alanine or methionine mutations at position 147 and suggesting that the interaction between LYS-147 and TYR-179 is important for the PmMOR reaction mechanism. Thus, the PmMOR results presented here suggest that TYR-179 and LYS-147 are involved in proton transfer similarly to the reduction mechanism hypothesized for progesterone reductase^24^.

As described above, comparisons of PmMOR and CrIS with the structure of Dl5ß–POR revealed that the residues contacting the citral/8-oxogeranial (PmMOR and CrIS) or progesterone (5ß–POR and PmMOR) substrate exhibit variability between the three enzymes. In order to test the role of selected residues in substrate specificity, a series of point mutations were made and activity with citral and progesterone were assayed (Table 3). Active site residues VAL-150, ILE-156, ILE-350, ALA-346 and PRO-353 from PmMOR were targeted for mutagenesis and mutated to the corresponding residue from Dl5ß–POR (V150M, I156Y, I350N and A346V). The P353F mutant expressed poorly and was not assayed. Single mutant V150M and double mutant V150M/I156Y resulted in increased specific activity of the PmMOR enzyme with respect to progesterone, with the mutant proteins accepting both citral and progesterone as substrate, with a clear preference for citral. The wild type PmMOR and A346V/I350N mutant had comparable specific activity against progesterone, similar to the Dl5ß–POR and lower activity with citral. Interestingly, 5ß–POR exhibited greater specific activity towards citral than to progesterone and the lowest specific activity to progesterone as compared to PmMOR and PmMOR mutants.

## Discussion

The structural studies and enzymatic assays presented here demonstrate that PmMOR is able to accommodate diverse substrates within its active site, to catalyse the reduction of aldehydes and carbon-carbon double bonds and to perform reductive cyclisation reactions to form the iridoid backbone. The crystal structures of CrIS and our structure of PmMOR provide the structural basis for reductive cyclisation activity and help to determine the key catalytic residues needed for efficient catalysis. We performed mutagenesis of the residues lining the substrate-binding pocket to investigate the different substrate preferences of iridoid synthases as compared to the highly related progesterone reductases. The structure-based mutagenesis results presented here suggest that increasing the specific activity of the enzyme is feasible through the addition of an acidic aspartic residue at the ASN-205 position. With all substrates tested this mutation resulted in increased turnover. Improving or switching substrate specificity, however, is not as straightforward as neither single nor double mutations dramatically improved specificity with respect to progesterone versus citral. This may be due to the simple reason that Dl5ß–POR accepts substrates other than progesterone and that mutating PmMOR residues to Dl5ß–POR residues would thus not result in improved specificity. Overall, PmMOR exhibits greater substrate promiscuity than CrIS, which is not able to turn over progesterone and higher activity towards progesterone than even annotated and well-studied progesterone reductases such as Dl5ß–POR.

PmMOR is able to accept citral, 8-oxogeranial and to a lesser extent progesterone as substrates. Interestingly, *P*. *major* does not have the cardenolide progesterone pathway, however the enzyme is able to accommodate this non-physiological substrate and perform the reduction reaction. A number of different plant species have annotated 5ß–PORs, but many of these lack the cardenolide pathway^6^. Based on the high degree of structural conservation in the SDR superfamily and the relative plasticity of the active site as shown here for PmMOR and Dl5ß–POR, it is likely that many annotated 5ß–POR’s have more promiscuous substrate activity which remains to be explored^2,34^. Indeed, our assays revealed that surprisingly Dl5ß–POR is more active with citral than with progesterone. This may also explain the extremely low turnover for progesterone of many putative 5ß–PORs (0.019 per second^35^ or 0.01 per second, this study) and warrants further inquiry, as few ISs have been identified but the iridoid pathway is much more common in plants than the cardenolide pathway^6^. As there are limited structures of SDR members in complex with substrate and a certain amount of ambiguity in the identity of true physiological substrates, determining which amino acid residues are important for substrate preference is still a difficult task. As we demonstrated here, the substrate preferences and full characterization of even apparently well-studied reductases such as the *D*. *lanata* 5ß–POR can reveal surprises, such as the ability to turn over compounds with highly different chemical scaffolds such as progesterone and citral. A deeper investigation of the activity of PRISE members will likely reveal additional potential substrates for these enzymes as we have shown for PmMOR.

While the enzymatic efficiency of PmMOR to produce nepetalactol from 8-oxogeranial is rather modest, it is comparable to the efficiency of the *C*. *roseus* iridoid synthase, with similar specific activity, but with a much higher *K*_m_ of 368 μM vs. 4 μM, for PmMOR and CrIS, respectively. The relatively poor substrate specificity may be due to PmMOR being an evolutionary snapshot with promiscuous activity towards a variety of different molecules and the ability to perform simple alkene reductions as well as reductive cyclisation. Based on ^1^H NMR characterization of the reaction of PmMOR with 8-oxogeranial, we could identify nepetalactol and observe a minor secondary product. Examination of the *P*. *major* genome reveals no other IS candidates based on homology to CrIS^22^. In addition, searching the EST *P*. *major* database identifies a homolog to the *C*. *roseus* upstream enzyme in the iridoid pathway, 8-hydroxygeraniol oxido-reductase. Thus, it is likely that iridoid biosynthetic pathway recently characterized in *C*. *roseus* is conserved in other species including *P*. *major* and that the PmMOR protein characterized here performs the reductive cyclisation reaction in the pathway. Whether the enzyme has additional functions *in planta* remains an open question to be explored.

Currently there are limited commercial sources of important iridoid natural products and few methods to produce complex iridoid derivatives synthetically. Plants hold the potential to be cost-effective “bioreactors” for the production of diverse chemical compounds. But this potential is hampered at the genetic level by poorly characterized biosynthetic pathways and at the protein level by poorly understood binding kinetics, substrate specificity determinants and catalytic mechanisms. One important challenge in optimizing secondary metabolic pathways for the production of therapeutic natural products is the ability to tune the activity of the enzymes involved with respect to catalytic efficiency, substrate specificity and product specificity. PmMOR, which demonstrates promiscuous substrate binding and reactivity, is an attractive potential candidate for protein engineering. Targeted or saturation mutagenesis of residues lining the active site in conjunction with substrate and product screening are envisaged for optimization of PmMOR for iridoid production, for example. The versatility of the enzyme in accepting different substrates potentially can be harnessed for enhanced production of a host of secondary metabolites, including iridoids, based in part on the enzymatic and structural studies presented here.

## Materials and Methods

### Protein expression and purification

The *PmMOR* gene was synthesized (GeneART, ThermoFisher Scientific) with codon usage optimized for *E*. *coli* overexpression (see Supplementary Fig. 1). The synthetic gene was cloned into the expression vector pESPRIT002^36,37^, using the AatII and NotI sites. The plasmid contains an N-terminal hexahistidine tag followed by a Tobacco Etch Virus (TEV) protease cleavage site. The plasmid was transformed into BL21 (DE3) cells, the cells were grown at 37 °C in lysogeny broth (LB) media supplemented with 50 μg/ml kanamycin until an optical density at 600 nm of 0.5, after which time the temperature was reduced to 22 °C. Protein expression was induced by the addition of 0.2 mM isopropyl β-D-1-thiogalactopyranoside (IPTG, Eurofins) and the cells were grown for 20 hours. Cells were harvested by centrifugation and resuspended with lysis buffer; 10 mM imidazole, 50 mM tris(hydroxymethyl)aminomethane (Tris, Sigma), pH 8.0, 100 mM NaCl, 1 mM tris(2-carboxyethyl) phosphine (TCEP, Sigma), 0.1% triton X-100 (Sigma) and 10% glycerol. Cells were disrupted with a Microfluidics Microfluidizer M-110L Fluid Processer and passed through a 5 mL Ni-NTA superflow Qiagen resin column. The imidazole concentration of the lysis buffer was increased to 20 mM to remove contaminants and the protein eluted with lysis buffer supplemented with 300 mM imidazole. The eluent was collected and buffer exchanged by dialysis (50 mM Tris pH 8.0, 100 mM NaCl, 10 mM imidazole, 1 mM TCEP) to reduce the imidazole concentration. The hexahistidine tag was cleaved with TEV protease (1:100) overnight and uncleaved protein and hexahistidine tagged TEV protease were removed by passage over the same Ni-NTA column. After concentrating to approximately 10 mg/mL, the protein was further purified over a Superdex 200 column (GE Healthcare) with 20 mM Tris, pH 8.0, 100 mM NaCl and 1 mM TCEP. Fractions of interest were pooled and the pure protein concentrated to approximately 16 mg/mL.

### Site-directed mutagenesis

All point mutants were made using Phusion high fidelity polymerase (NEB) according to the manufacturer’s protocol. All primers were purchased from Eurofins and used without further purification. The pESPRIT002 expression plasmid containing wild type PmMOR was used as a template. Double mutants V150M/I156Y and A346V/I350N were made sequentially with the first mutation acting as a template (i.e. V150M or A346V) for the second mutation. PCR was performed in a Mastercycler gradient (Eppendorf, Hamburg, Germany) with a single cycle of 90 s, 98 °C, 30 s, 50 °C, 5 min, 72 °C followed by 25 cycles of 30 s, 98 °C, 30 s, 50 °C, 5 min, 72 °C and a single cycle of 10 min at 72 °C (final extension). Mutations were confirmed by sequencing (MWG AG, Germany), and the proteins were produced in *E*. *coli*, BL21(DE3), as described above for the wild type protein. Protein was obtained of constructs N205A, N205D, K147A, V150M, V150M/I156Y, A346V/I350N and K147M/N205D.

### Enzymatic assays

Assays were carried out in a total volume of 200 µL with 20 mM MOPS, pH 7.0, 150 µM NADPH, 6 µL DMSO and 10–3000 µM substrate (8-oxogeranial, citral or progesterone). 8-oxogeranial was synthesized according to the published protocols^4^, citral and progesterone were purchased from Sigma. Substrate concentration was varied between 10 µM and 3000 µM for citral and 8-oxogeranial and 10 µM and 200 µM for progesterone. The progesterone concentration was not increased above 200 µM due to its limited solubility in aqueous buffer. For determination of kinetic constants for the wild type enzymes (PmMOR and 5ß–POR) substrate concentrations were varied. Specific activity assays were performed using a substrate concentration of 1 μM and 0.1 μg of protein, well above the *K*_*m*_ as determined for the wild type proteins. The reaction was monitored by the reduction in absorbance of NADPH at 340 nm and measured using a Shimadzu UV-VIS recording spectrophotometer according to Geu-Flores^4^. All assays were performed in quadruplicate. Results were analysed using Kaleidagraph software.

### Crystallization and data collection

The protein was crystallized using the hanging drop vapour diffusion method. Protein (wild type or PmMOR^V150M^) at 16 mg/mL was pre-incubated for approximately 1 h with 1–5 mM NADP^+^ and either 5 mM citral or 8-oxogeranial or a saturated solution of progesterone (<5 mM). The reservoir conditions for wild type/NADP^+^/8-oxogeranial, PmMOR^V150M^/NADP^+^/progesterone and PmMOR^V150M^/NADP^+^/citral were 0.1 M Bis-Tris Propane, 5% glycerol, and 16.5% to 19% PEG 5000 MME. For the wild type PmMOR/NAD^+^/progesterone, crystals were obtained in 0.1 M sodium acetate pH 4.6, 0.2 M ammonium acetate and 30% PEG 4000. Glycerol (15%) was added directly to the drops as a cryoprotectant, the crystals were harvested and flash frozen in liquid nitrogen.

### Data collection, processing, and refinement

All datasets were collected at 100 K on beamline ID23–2 of the ESRF, Grenoble, France. Indexing was performed using EDNA^38^ and the default optimized oscillation range and collection parameters used for data collection. The data sets were integrated and scaled using the programs *XDS* and *XSCALE*^39,40^. Molecular replacement was performed using PHASER^41^ with the structure of the *D*. *lanata* 5ß–POR as a search model (PDB 2V6F) for the PmMOR apo dataset. All subsequent datasets were phased using the PmMOR apo structure as a molecular replacement model. All refinements were performed using BUSTER^42^ and PHENIX^43^. The structures are deposited under PDB codes 5MLH, 5MLR, 5MLM and 6GSD for PmMOR/NADP^+^/8-oxogeranial, PmMOR^V150M^/NADP^+^/citral, PmMOR^V150M^/NADP^+^/progesterone and PmMOR/NAD^+^/progesterone, respectively. Omit maps were produced by deleting the ligand, followed by phenix.dynamics to introduce random coordinate shifts followed by refinement in BUSTER.

### Thin Layer Chromatography

Enzyme reactions were monitored using TLC assays and R_f_ values compared to starting material and product. In a typical assay, a 200 µL reaction volume with 20 mM MOPS, pH 7.0, 150 µM NADPH, 3000 µM 8-oxogeranial, 3000 µM citral or 200 µM progesterone and 10 µg protein was incubated at room temperature. Protein used was wild type or mutant PmMOR; *C*. *roseus* iridoid synthase (CrIS) (gift from Sarah O’Connor, John Innes Center) or *D*. *lanata* progesterone reductase (gift from Wolfgang Kreis, Friedrich-Alexander-Universitat Erlangen-Nurnberg, Germany). Reactions were left for 24 h and then stopped by the addition of 200 µL dichloromethane. The mixture was vortexed and the organic layer collected. The organic layer was spotted onto a silica TLC plate along with substrate and product as controls. The plates were run with a mix of 7:3 hexanes:ethyl acetate. The plates were developed with anisaldehyde stain (3.5% sulfuric acid, 2.6% p-anisaldehyde, 1.1% glacial acetic acid, 93% ethanol). R_f_ values for 8-oxogeranial, citral, progesterone, nepetalactol (8-oxogeranial reaction product), citronellal (citral reaction product) and 5ß-dihydroprogesterone (progesterone reaction product) were 0.36, 0.32, 0.35, 0.78, 0.64 and 0.5, respectively.

### Nuclear Magnetic Resonance Imaging (NMR)

A large-scale reaction using the buffer conditions described above and approximately 100 µg of PmMOR and 3 mM 8-oxogeranial in a 1 mL volume was run as per^4^. Aliquots of the reaction mixture were taken at 1 h, 3 h and 24 h after which the reaction was stopped by the addition of methylene chloride. The organic layer was isolated via extraction, dried with sodium sulfate, filtered and concentrated. The residue was dissolved in 200 µL of deuterated chloroform for ^1^H NMR analysis. The crude extract showed a mixture of starting material, 8-oxogeranial, and product, nepetalactol. No product peaks were seen in the 1 h reaction and very small product peaks were noted in the 3 h reaction (corresponding to less than 1% of starting material conversion). The chemical shifts of the 8-oxogeranial showed characteristic olefin peaks at 6.4 (triplet) and 5.9 (doublet) and aldehyde peaks at 9.4 and 10.3. The cyclised product had a single olefin peak at 6.0 (singlet) and a peak for the lactol C-H at 4.8 with a smaller hydroxyl peak at 5.2, indicative of a mix of diastereomers in the product. The amount of product formed was estimated based on the integration of the product nepetalactol peaks as compared to the starting 8-oxogeranial peak. After an overnight incubation, approximately 20% of the starting material was converted to product.

## Electronic supplementary material

Supplementary Information

## Data Availability

All structural data has been deposited in the PDB (5MLH, 5MLR, 5MLM and 6GSD) and is available.
