# Supplementary material for: A multisubstrate reductase from Plantago major: structure-function in the short chain reductase superfamily
Source: Sci Rep. 2018 Oct 4;8:14796. doi: 10.1038/s41598-018-32967-1 (PMC6172241; doi:10.1038/s41598-018-32967-1)
Supplement: Supplementary file 1 — Supplementary Information [file 41598_2018_32967_MOESM1_ESM.docx]

**Supplemental Information**

A multisubstrate reductase from *Plantago major*: structure-function in the short chain reductase superfamily

Rachel Fellows^a^, Christopher M. Russo^b^, Catarina S. Silva^a^, Soon Goo Lee^c^, Joseph M. Jez ^c^, John D. Chisholm^b^, Chloe Zubieta^d,f^, Max Nanao^a,e,f^

**Supplemental Information**

**Supplementary Figure 1.**

DNA and amino acid sequence of PmMOR including an N-terminal 6x-Histidine tag and TEV protease cleavage site.

DNA sequence of *PmMOR* after codon optimisation for *E. coli* expression

CATATGGGCCACCATCATCACCACCATGATTATGATATTCCAACTACCGAGAATTTGTAT

TTTCAGGGAAGCAGCGTTGCACTGATTGTTGGTGTTACCGGTATTGTTGGTAATAGCCTG

GCAGAAATTCTGCCGCTGGCAGATACCCCGAGCGGTCCGTGGAAAGTTTATGGTGTTGCA

CGTCGTCCGCGTCCGGCATGGAATGAAGATAATCCGATTAACTATATCCGCTGCGATATT

AGCGATCCGAAAGATACCCAAGAAAAACTGAGTCCGCTGACCGATATCACCCATGTTTTT

TATGTGACCTGGGCAAATCGTAGCACCGAAGTTGAACGTTGTGAAGCAAATGGTAAGATG

CTGAAAAATGTGCTGGATGTGGTGATTCCGAATTGTCCGGATCTGAAACACATTAGCCTG

CAGACCGGTCGTAAACATTATGTTGGTCCGTTTGAACTGATCGGCAAAATTGAAACCCAT

GATCCGCCTTTTACCGAAGATCTGCCTCGTCTGAAATTCGATAACTTCTATTATACCCAA

GAGGACCTGCTGTTTGAAGAAGTGGAAAAAAAAGAAGGCCTGACCTGGTCAGTTCATCGT

CCGGGTAACATTTTTGGTTTTAGCCCGTATAGCATGATGAATCTGGTTGGCACCCTGTGT

GTTTATGCAGCAATTTGTAAACATGAAGGTAAAGTGCTGCGTTTTCCGGGTTGTAAAGCA

GCATGGGATGGTTATAGCGATTGTAGTGATGCAGATCTGATTGCCGAACATCATATTTGG

GCAGCAGTTGATCCGTATGCAAAAAATGAAGCCTTTAATGTGAGCAACGGCGACGTGTTC

AAATGGAAACATTTTTGGAAAGTTCTGGCCGAACAGTTTGGTGTTGAATGTGGTGAATAT

GAAGAAGGCGAAAATCTGAAACTGCAGGATCTGATGAAAGGTAAAGAACCGGTTTGGGAA

GAAATTGTGCGTGAAAATGGTCTGGCAAGCACCAATCTGGAAGATGTTGCAGTTTGGTGG

TTTTCAGATGCAGTTCTGGATATTCCGTGTCCGCTGGATAGCATGAATAAAAGCAAAGAA

CATGGCTTTCTGGGCTTTCGCAATAGCAAAAATAGCTTTATTAGCTGGATTGATAAAGCC

AAAGCCTACAAAATTGTGCCGTAAGCGGCCGC

Parent amino acid sequence

MGHHHHHHDY DIPTTENLYF QGMSWWWAGA IGAAKKRSDE

DEALPKHSSV ALIVGVTGIV GNSLAEILPL ADTPSGPWKV

YGVARRPRPA WNEDNPINYI RCDISDPKDT QEKLSPLTDI

THVFYVTWAN RSTEVERCEA NGKMLKNVLD VVIPNCPDLK

HISLQTGRKH YVGPFELIGK IETHDPPFTE DLPRLKFDNF

YYTQEDLLFE EVEKKEGLTW SVHRPGNIFG FSPYSMMNLV

GTLCVYAAIC KHEGKVLRFP GCKAAWDGYS DCSDADLIAE

HHIWAAVDPY AKNEAFNVSN GDVFKWKHFW KVLAEQFGVE

CGEYEEGENL KLQDLMKGKE PVWEEIVREN GLASTNLEDV

AVWWFSDAVL DIPCPLDSMN KSKEHGFLGF RNSKNSFISW

IDKAKAYKIV P
